# Gamma-Herpesvirus Latency Requires T Cell Evasion during Episome Maintenance

**DOI:** 10.1371/journal.pbio.0030120

**Published:** 2005-03-22

**Authors:** Neil J Bennett, Janet S May, Philip G Stevenson

**Affiliations:** **1**Division of Virology, Department of PathologyUniversity of CambridgeUnited Kingdom; University of Wisconsin–MadisonUnited States of America

## Abstract

The gamma-herpesviruses persist as latent episomes in a dynamic lymphocyte pool. Their consequent need to express a viral episome maintenance protein presents a potential immune target. The glycine–alanine repeat of the Epstein–Barr virus episome maintenance protein, EBNA-1, limits EBNA-1 epitope presentation to CD8^+^ T lymphocytes (CTLs). However, CTL recognition occurs in vitro, so the significance of such evasion for viral fitness is unclear. We used the murine gamma-herpesvirus-68 (MHV-68) to define the in vivo contribution of *cis*-acting CTL evasion to host colonisation. Although the ORF73 episome maintenance protein of MHV-68 lacks a glycine–alanine repeat, it was equivalent to EBNA-1 in conferring limited presentation on linked epitopes. This was associated with reduced protein synthesis and reduced protein degradation. We bypassed the *cis-*acting evasion of ORF73 by using an internal ribosome entry site to express in *trans*-a CTL target from the same mRNA. This led to a severe, MHC class I–restricted and CTL-dependent reduction in viral latency. Thus, despite MHV-68 encoding at least two *trans*-acting CTL evasion proteins, *cis-*acting evasion during episome maintenance was essential for normal host colonisation.

## Abstract 2

The mouse gamma-herpesvirus is invisible to its host immune system by virtue of its episome maintenance protein (ORF73), which is analogous to a protein in the human EBV

## Introduction

Latent herpesviruses can persist as episomes in quiescent cells without viral protein expression. However, the gamma-herpesviruses are characteristically latent in memory lymphocytes, which intermittently divide. Viral genomes must therefore be replicated and segregated between daughter cells in step with cellular mitosis. This requires a viral episome maintenance protein, creating a potential target for the immune recognition of latently infected cells. A glycine–alanine repeat in the Epstein–Barr virus (EBV) episome maintenance protein, EBNA-1 [1], inhibits its degradation [2] and translation [3] such that EBNA-1 epitopes are poorly presented to CD8^+^ T lymphocytes (CTLs) [4,5,6]. However, the in vivo effectiveness of the glycine–alanine repeat and its quantitative contribution to host colonisation remain unknown. In vitro studies have suggested that abortive EBNA-1 translation products still provide sufficient epitopes for EBNA-1-specific CTLs to recognise latently infected B cells [7,8,9].

In vivo immune function is difficult to analyse directly with human viruses. However, the murid pathogen murine gamma-herpesvirus-68 (MHV-68) affords an opportunity to manipulate a gamma-herpesvirus in its natural host. MHV-68 is a gamma-2-herpesvirus [10], more closely related to the Kaposi's sarcoma–associated herpesvirus than to EBV [11], but clear functional parallels exist between all three viruses. Like EBV, MHV-68 causes an acute infectious mononucleosis-like illness, associated with a massive expansion of latently infected germinal centre B cells, and it persists in memory B cells [12,13,14]. The episome maintenance protein of gamma-2-herpesviruses is encoded by ORF73 [15]. Just as EBNA-1-deficient EBV [16] and ORF73-deficient Kaposi's sarcoma–associated herpesvirus [17] fail to maintain latency in vitro, ORF73-deficient MHV-68 has a profound latency deficit in vivo [18,19].

At least two MHV-68 gene products inhibit CTL recognition of latently infected cells. The M3 chemokine-binding protein [20,21] is abundantly secreted by lytic virus [22]. This co-exists with latent virus in infected lymphoid tissue [14,23,24]. Bystander protection by M3 [25] probably explains why M3 disruption causes mainly a reduction in MHV-68 latency amplification [26,27]. Protection by M3 may be quite context-dependent [28] and probably functions best in acute infection, before M3-specific immunity is established. A second MHV-68 CTL evasion protein, K3, degrades major histocompatibility complex (MHC) class I heavy chains [29] and TAP (transporter associated with antigen processing) [30]. K3 is transcribed in latency as well as in the viral lytic cycle, and again protects against CTLs during latency amplification [31]. However, K3 is not expressed in all forms of latency: it is not detectable in the MHV-68-infected S11 tumor line [32] or in persistently infected, B cell–deficient mice [33]. Notably, ORF73 disruption causes a much more profound latency deficit than does K3 disruption, implying that immune evasion by K3 is not important in every cell expressing ORF73. These restrictions on M3 and K3 function imply that further immune evasion mechanisms contribute to MHV-68 latency.

Selective EBNA-1 expression occurs in normal EBV persistence [34,35] and is characteristic of EBV-associated Burkitt's lymphoma [36]. The gamma-2-herpesviruses presumably implement an equivalent program of selective ORF73 expression. We have used MHV-68 to determine the in vivo importance of avoiding epitope presentation during episome maintenance. We first established that the MHV-68 ORF73 is equivalent to EBNA-1 in reducing the presentation of an MHC class I–restricted epitope linked to it in *cis.* We then modified the ORF73 transcript to bypass this evasion, and used the mutant virus to define the consequences of epitope presentation for viral fitness. Our analysis of MHV-68 implies that if MHC class I–restricted viral epitope presentation occurs during gamma-herpesvirus episome maintenance, even a relatively small CTL response can very effectively clear latent infection.

## Results

### Limited Presentation of a CD8^+^ T Cell Epitope Linked in *cis* to ORF73

As with EBNA-1, CTL epitopes in the MHV-68 ORF73 have been hard to find, perhaps reflecting limited ORF73 entry into the MHC class I antigen-processing pathway. To determine whether ORF73 is similar to EBNA-1 in its resistance to MHC class I–restricted antigen presentation, we introduced the H2-K^b^-restricted SIINFEKL epitope of ovalbumin (OVA) near the ORF73 C-terminus (73-SC) or N-terminus (73-SN). L929-K^b^ cells transfected with 73-SC or 73-SN were poorly recognised by the SIINFEKL-specific hybridoma, B3Z (Figure 1A), suggesting poor antigen processing. There was no evidence for ORF73 inhibiting SIINFEKL presentation from co-transfected OVA (Figure 1B). The apparent immune evasion therefore acted in *cis* rather than in *trans,* and it was not due to any ORF73 toxicity.

**Figure 1:**
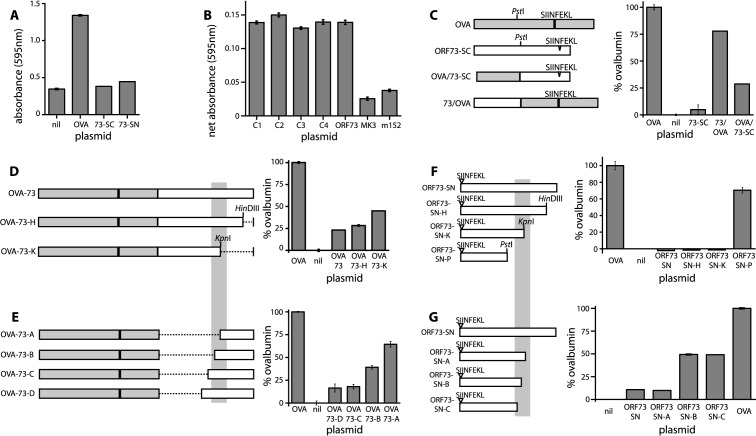
Inhibition of MHC Class I–Restricted Epitope Presentation by Physical Linkage to ORF73 (A) The SIINFEKL epitope of OVA was introduced into ORF73 near either its N-terminus (ORF73-NC) or its C-terminus (ORF73-SC). Both ORF73 derivatives were cloned into the pcDNA3 expression vector and compared with OVA in the same vector for their capacity to stimulate the SIINFEKL-specific T cell hybridoma B3Z after transfection into L929-K^b^ cells. After 48 h, beta-galactosidase production was assayed by cell lysis in the presence of chlorophenol-red-beta-D-galactoside and reading absorbance at 595 nm. nil, vector only. (B) L929-K^b^ cells were co-transfected with OVA plus the plasmid indicated. C1–C4 are control plasmids, expressing MHV-68 ORFs 19, 30, 31, and 35, respectively. K3 degrades MHC class I heavy chains and m152 is a murine cytomegalovirus gene that retains MHC class I molecules in the endoplasmic reticulum. Net absorbance = A_595_ with co-transfection − A_595_ with untransfected cells (<0.02). (C) Hybrids of OVA and ORF73-SC were made to identify regions of ORF73 that inhibited SIINFEKL presentation. Responses are expressed as 100(A_595_ with plasmid − A_595_ with untransfected)/(A_595_ with OVA transfection − A_595_ with untransfected). nil, vector only. Mean ± standard deviation (SD) values of triplicate cultures are shown. Each graph is representative of at least three separate experiments. In at least one experiment, equal transfection efficiency was confirmed by co-transfecting a GFP expression plasmid and checking fluorescence under ultraviolet illumination. (D) ORF73 was fused to the C-terminus of the OVA coding sequence in pcDNA3. C-terminal deletions were then made as shown. Each construct was transfected into L929-H2-K^b^ cells. The shaded area in (D–G) highlights a region of ORF73 that appeared to be important for inhibiting epitope presentation. (E) N-terminal ORF73 truncations were generated by PCR and fused in frame to amino acid 325 of OVA. Each construct was transfected into L929-H2-K^b^ cells and assayed for SIINFEKL presentation as in (D). (F) Progressive truncations of ORF73-SN were assayed for their capacity to present the SIINFEKL epitope to B3Z cells after transfection into L929-K^b^ cells. Selective presentation from the ORF73-SN-PstI construct was confirmed in multiple experiments, including independent plasmid preparations. (G) PCR-generated C-terminal truncations of ORF73-SN were assayed for SIINFEKL presentation after transfection of L929-H2-K^b^ cells. Deletions across the area identified as important for inhibiting epitope presentation in (D–E) again improved epitope presentation.

To identify possible contributions of the N-terminal and C-terminal regions of ORF73 to its poor epitope presentation, we made hybrids of OVA (amino acid residues 1–325) and 73-SC using a shared PstI site (Figure 1C). N-terminal ORF73 (amino acid residues 1–150) diminished SIINFEKL presentation from OVA relatively little, suggesting that any inhibitory segment was C-terminal of its PstI site (amino acid residues 151–314). With N-terminal OVA, SIINFEKL presentation from 73-SC remained low.

### A Key Region of ORF73 for *cis-*Acting Immune Evasion

We adopted two strategies to localise an inhibitory segment C-terminal of the ORF73 PstI site. First, we fused the entire ORF73 coding sequence to the C-terminus of OVA, thereby inhibiting SIINFEKL presentation from OVA. C-terminal truncation of the fusion protein (Figure 1D) up to the ORF73 HinDIII site (residue 278) then had little effect, but truncation up the ORF73 KpnI site (residue 206) restored SIINFEKL presentation somewhat. N-terminal truncation (Figure 1E) to ORF73 residue 166 (OVA-73C) did not compromise the inhibition, but further truncation to residue 186 (OVA-73B) and then residue 206 (OVA-73A) progressively improved SIINFEKL presentation. These data were consistent with the region of ORF73 encoding approximately residues 170–220 (the shaded region in Figure 1D–1G) reducing antigen presentation.

We also truncated 73-SN from its C-terminus and looked for presentation of its N-terminal SIINFEKL epitope (Figure 1F). With SIINFEKL in this context, the same C-terminal truncations as in Figure 1D (amino acid residues 1–204) gave no epitope presentation, presumably because SIINFEKL was less efficiently processed from 73-SN than it was from OVA. However, a further truncation up to the ORF73 PstI site (residue 150) dramatically improved SIINFEKL presentation. PCR-generated C-terminal truncations of 73-SN (Figure 1G) supported the idea of a region just upstream of the ORF73 KpnI site (ORF73-SN-B) limiting the presentation of SIINFEKL from 73-SN. These results were therefore consistent with those shown in Figure 1D and 1E.

### The Effects of ORF73 on the Turnover of Linked OVA

The inhibition of EBNA-1 epitope presentation by its glycine–alanine repeat has been attributed principally to reduced protein synthesis and secondarily to reduced protein degradation [3]. We therefore analysed the effect of ORF73 on OVA turnover using constructs equivalent to those in Figure 1E, except that we removed the signal sequence of OVA to avoid any protein secretion (SOVA, Figure 2A). All constructs were cloned into pcDNA3 and transfected into 293T cells. We observed a hierarchy of SOVA/ORF73 antigen presentation (Figure 2B) similar to that seen with the OVA/ORF73 hybrids: amino acids 206–314 of ORF73 (SOVA-73A) reduced somewhat SIINFEKL presentation from SOVA; amino acids 166–314 (SOVA-73C) reduced it further; including an additional 40 amino acids of ORF73 (SOVA-73E) gave no additional inhibition.

**Figure 2:**
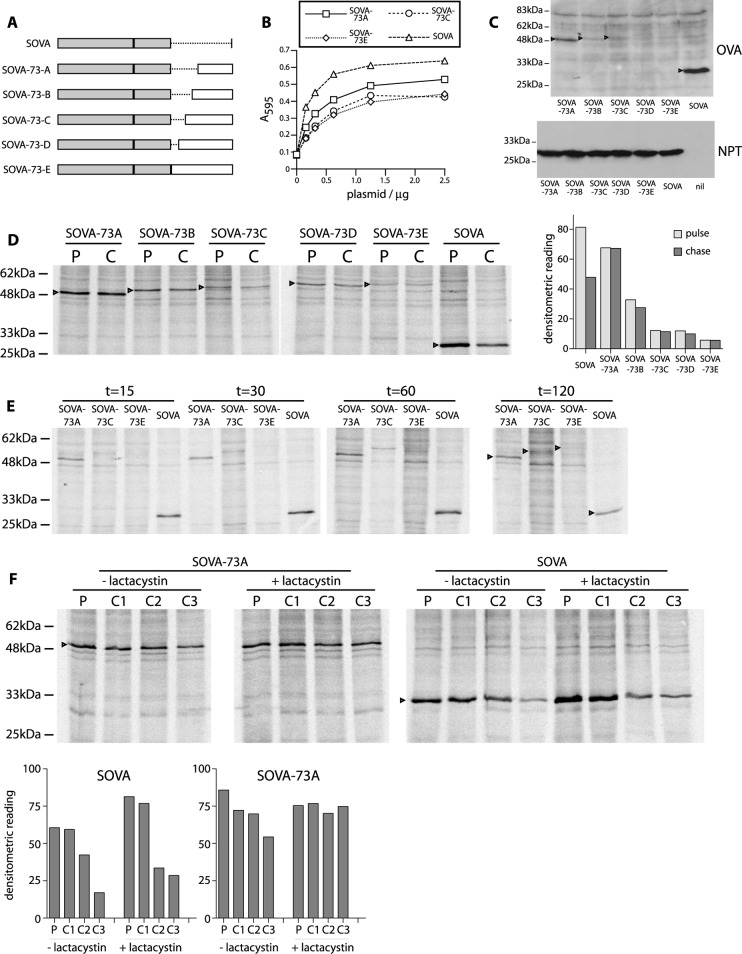
Inhibition of Epitope Presentation by ORF73 Fusion to OVA Correlates with Reduced Translation of the Fusion Protein (A) N-terminal ORF73 truncations equivalent to those in Figure 1E were fused in frame to the C-terminus of OVA amino acids 41–325, thereby removing both the OVA signal sequence and the ORF73 nuclear localisation signal. (B) Serial dilutions of an expression plasmid containing each fusion gene were transfected into L929-H2-K^b^ cells as in Figure 1. SIINFEKL presentation was assayed using beta-galactosidase production from the B3Z hybridoma. (C) Equivalent transfected cells were immunoblotted with an anti-OVA rabbit serum (OVA). Fusion products are indicated by arrowheads where visible. Parallel immunoblots for neomycin phosphotransferase II (NPT), which is expressed from a different promoter of the same plasmid (pcDNA3), were used to control for transfection efficiency. The endogenous neomycin phosphotransferase II expressed by 293T cells was not visible at this exposure. One of three equivalent experiments is shown. (D) Forty-eight hours after transfection with the constructs indicated, 293T cells were pulse-labelled (P) for 30 min with ^35^S-cysteine/methionine, followed by a 2-h chase (C) with excess unlabelled cysteine/methionine. OVA derivatives were then immunoprecipitated with an OVA-specific rabbit serum and resolved by SDS-PAGE. The specific bands corresponding to each fusion protein are indicated by arrowheads. The graph shows densitometry readings for each band. (E) 293T cells transfected with selected fusion proteins were labelled for a variable period (15–120 min) as indicated. OVA derivatives were then immunoprecipitated and analysed as in (D). Arrowheads show the predicted position of the relevant fusion proteins for the 120-min label samples. (F) Either SOVA-ORF73A or SOVA was transfected into 293T cells. Forty-eight hours later the cells were pulse-labelled (P) for 15 min with ^35^S-cysteine/methionine, followed by a 15-min (C1), 45-min (C2), and 105-min (C3) chase with excess unlabelled cysteine/methionine. This was done in the presence or absence of 100 μM lactacystin. The graph shows densitometry readings for each specific band.

Steady-state protein levels, determined by immunoblotting transfected cell lysates (Figure 2C), were greatest with SOVA, followed by SOVA-73A, and least with SOVA-73D-E. Parallel immunoblots for neomycin phosphotransferase II, expressed from a different promoter of the same plasmid, showed no significant variation in signal, arguing against an effect of ORF73 on cell viability or transfection efficiency. Pulse-chase metabolic labelling of transfected 293T cells and immunoprecipitation with an OVA-specific antiserum (Figure 2D) showed that all the ORF73/SOVA fusions were more stable than SOVA alone, and that their labelling was reduced as more ORF73 sequence was attached. The differences in protein synthesis rate (Figure 2D) correlated with steady-state protein levels (see Figure 1C). Using a variable labelling window (Figure 2E), SOVA and SOVA-73A were detectable after a 15-min pulse, whereas SOVA-73E was hard to discern even after a 120-min pulse.

The apparent stability conferred by ORF73 on SOVA (Figure 2D) was confirmed by further analysis of SOVA-73A, the fusion protein that labelled most efficiently (Figure 2F). After a 20-min pulse, most labelled SOVA was lost over the next 2 h, whereas the labelled SOVA-73A was relatively well maintained. Proteasome inhibition with lactacystin partially stabilised SOVA, but it remained less stable than SOVA-73A. Thus, the stability afforded by amino acids 206–314 of ORF73 appeared to extend beyond protection against proteasome-mediated degradation. Overall, the *cis-*acting immune evasion of ORF73 appeared functionally similar to that of EBNA-1, in that reduced epitope presentation was associated with reduced protein synthesis and reduced protein degradation. In contrast to EBNA-1, these functions were mediated by distinct regions of ORF73. The key region for inhibiting epitope presentation (see Figure 1D–1G) corresponded to that responsible for reducing protein synthesis.

### An MHV-68 Mutant That Lacks *cis-*Acting Immune Evasion

The region of ORF73 responsible for *cis-*acting immune evasion—that encoding amino acids 170–220—shows considerable amino acid homology to both EBNA-1 and the Kaposi's sarcoma–associated herpesvirus ORF73 [37]. Any mutagenesis was therefore likely to compromise other ORF73 functions. Also, altering this region would change the steady-state levels of ORF73, with likely toxic effects in latently infected cells. In order to keep ORF73 function intact, therefore, we bypassed the *cis-*acting immune evasion not by mutating the ORF73 protein, but by modifying its mRNA. Thus, we inserted an internal ribosome entry site (IRES) just downstream of the ORF73 coding region and used this to co-express either green fluorescent protein (GFP) or three tandem MHC class I–binding peptides (Figure 3A).

**Figure 3:**
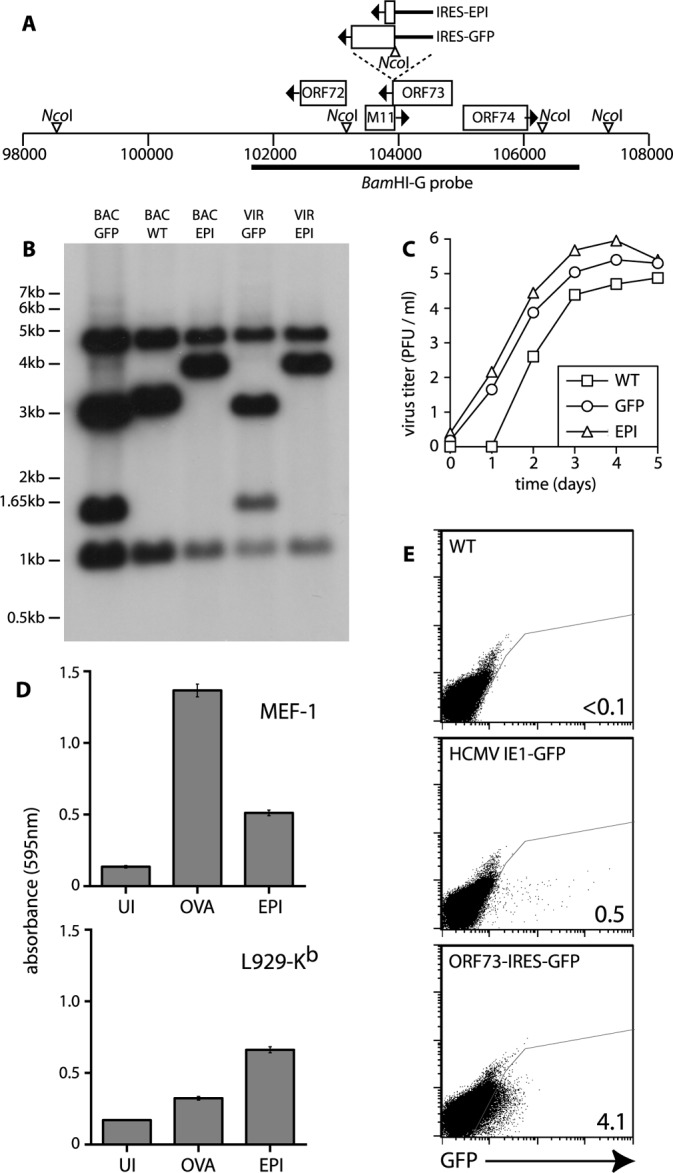
Modification of the MHV-68 Genome to Overcome *cis-*Acting Immune Evasion by ORF73 (A) An IRES element was inserted just downstream of ORF73, between its stop codon and that of M11. This allowed either three tandem CD8^+^ T cell epitopes (EPI) or GFP to be translated from the ORF73 mRNA. (B) DNA from BAC-cloned viral genomes (BAC) or virus-infected cells (VIR) was digested with NcoI, electrophoresed, transferred to nylon membranes, and blotted with a probe corresponding to the BamHI-G genomic fragment shown in (A). The predicted bands for WT virus were 1,021 bp, 3,121 bp, and 4,630 bp. The IRES-GFP insert introduced an NcoI site such that the WT 3,121-bp band was cut into 2,975-bp and 1,466-bp fragments. The NcoI site was lost from the IRES-EPI insert, such that the WT 3,121-bp band became a 3,861-bp band. (C) BHK-21 cells were infected (0.01 PFU/cell) with WT, GFP, or EPI viruses as indicated. Plaque titres of cell cultures are shown with time after infection. (D) H2^b^ MEF-1 cells or L929-K^b^ cells were left uninfected (UI) or infected for 2 h with MHV-68 expressing either OVA under a strong lytic promoter (OVA) or the SIINFEKL epitope of OVA as part of the ORF73-IRES-EPI construct (EPI). B3Z cells were then added, and 18 h later their beta-galactosidase response was assayed using chlorophenol-red-beta-D-galactoside substrate. Mean ± SD values of triplicate cultures are shown. The data are from one or two equivalent experiments. (E) A20-syndecan-1 cells were infected (20 PFU/cell) with GFP^−^ WT virus, WT virus with an HCMV IE1 promoter-driven GFP expression cassette (HCMV IE1-GFP), or with the ORF73-IRES-GFP virus. The numbers indicate the percentage of total cells in the gated region (GFP^+^). Expression from the HCMV IE1 promoter is probably limited to lytic infection, whereas ORF73 is expressed in latency.

Southern blots confirmed the predicted genomic structure of the ORF73-IRES-epitope (EPI) and ORF73-IRES-GFP viruses (Figure 3B). Both mutants showed unimpaired growth in vitro (Figure 3C). Infection of H2-K^b^ fibroblasts with the EPI virus established that its SIINFEKL epitope could be processed and presented (Figure 3D). MHV-68 expressing OVA from an intergenic expression cassette under the control of an ectopic viral M3 promoter (MHV-OVA), which shows high-level lytic cycle OVA production (data not shown), was tested in parallel. In murine embryonic fibroblast (MEF)-1 cells, which support MHV-68 lytic replication, MHV-OVA showed better SIINFEKL presentation than did the EPI virus. In L929 cells, which support viral entry into the lytic cycle relatively poorly [38], the EPI virus gave better SIINFEKL presentation than did MHV-OVA. These data were consistent with the EPI virus presenting SIINFEKL in latency.

The GFP mutant provided further evidence that the IRES constructs were expressed in latency. Although MHV-68 is predominantly latent in B cells in vivo, it appears to infect B cells poorly in vitro. This may reflect that efficient infection by MHV-68 virions requires cell-surface glycosaminoglycans [39]. We therefore enhanced infection of the A20 B cell line by transducing it with a retroviral vector expressing the extracellular domain of syndecan-1, a major carrier of cell-surface glycosaminoglycans [40], linked to the transmembrane and cytoplasmic domains of H2-D^b^. This form of syndecan-1 resists proteolytic cleavage. GFP expressed from a human cytomegalovirus IE-1 promoter [41] gave green fluorescence in relatively few A20-syndecan-1 cells. Although the ORF73-IRES-GFP virus gave much weaker fluorescence, many more cells were positive (Figure 3E). These data were consistent with B cell infection being predominantly latent, and with gene expression from the IRES constructs in latency.

### Epitope Presentation during Episome Maintenance Leads to a Severe In Vivo Latency Deficit

We tested the capacity of the EPI virus to replicate in vivo by intranasal infection of C57BL/6J mice (Figure 4). There was no difference between wild-type (WT) and EPI viruses in lytic replication in lung epithelial cells or in seeding latent virus to the spleen (Figure 4A). However, by 14 d after infection, when WT virus had reached its peak latent load, the titre of EPI virus was drastically reduced (Figure 4B). In agreement with the reduced number of infectious centres, the EPI virus genome load was low (Figure 4C) and there was little virus-driven B cell activation, T cell activation, or Vbeta4^+^CD8^+^ T cell expansion (Figure 4D). The GFP control virus showed no such deficit, so it was not the IRES element that compromised ORF73 function. An independently derived EPI mutant showed a similar in vivo latency deficit, and reverting the EPI mutation restored latency establishment to normal levels (Figure 4E). The latency deficit was therefore due specifically to the expression of a poly-epitope construct downstream of ORF73.

**Figure 4:**
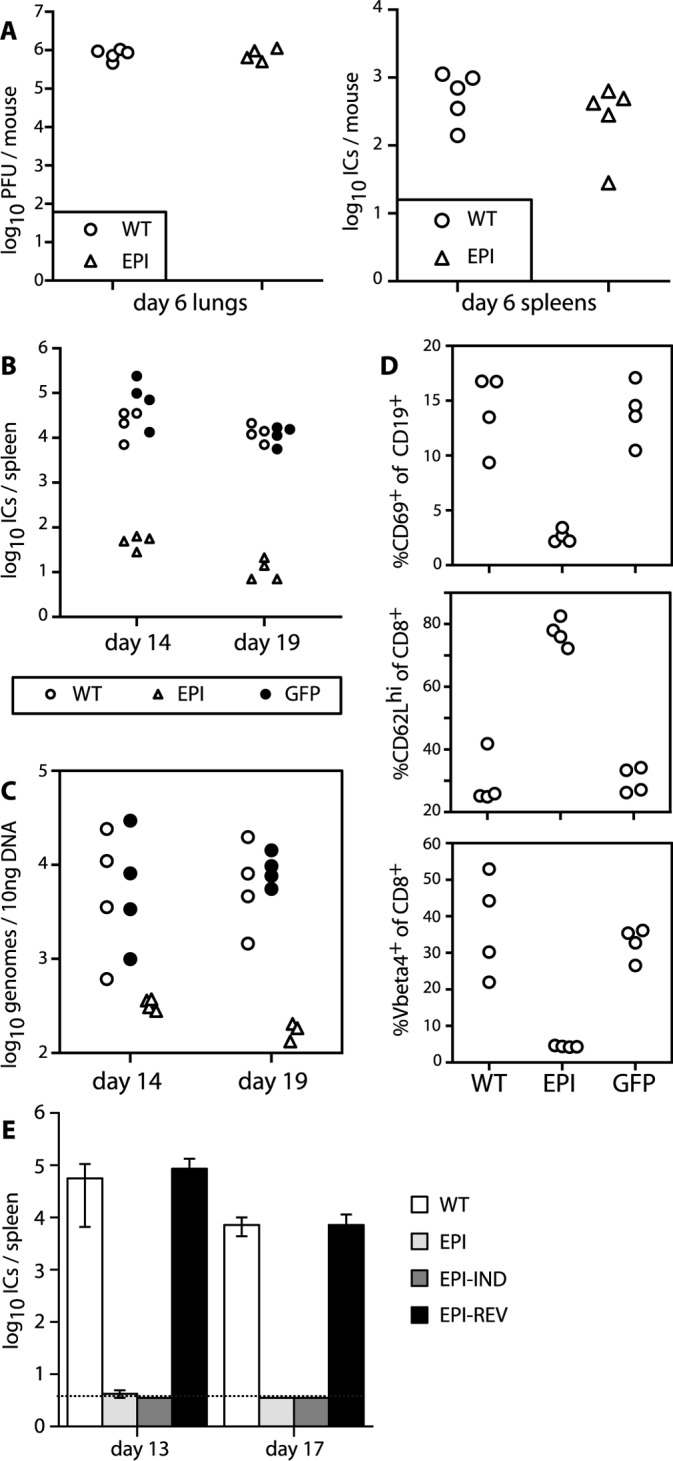
Replication of the IRES-EPI Virus In Vivo (A) Six days after intranasal infection with WT or EPI viruses as indicated, infectious virus in lungs was titred by plaque assay (left panel) and infectious plus latent virus in spleens was titred by infectious centre assay (right panel). Each point shows an individual mouse. Pre-formed, infectious virus was undetectable in equivalent, freeze-thawed spleen samples, so the infectious centres represent latent virus. (B) By 14 d post-infection, infectious centre titres were much lower with the EPI virus than with WT. The GFP control virus is shown for comparison. This difference was preserved at day 19 post-infection, indicating that the EPI virus was not merely delayed in host colonisation. (C) DNA was extracted from spleens and its viral genome content quantitated by real-time PCR. Genome loads broadly reflected the infectious centre titres, indicating that the viral load was reduced rather than the efficiency of ex vivo reactivation. (D) As a further measure of host colonisation, we measured B cell activation (CD69 expression on CD19^+^ B cells) at 14 d post-infection and CD8^+^ T cell activation (loss of CD62L expression) at 19 d post-infection. We also measured the day 19 expansion of the Vbeta4^+^CD8^+^ T cell subset that is characteristic of MHV-68-associated infectious mononucleosis. All these measures correlated closely with the viral latent load in lymphoid tissue and were markedly reduced with the EPI virus compared to WT or GFP. GFP expression was undetectable in ex vivo B cells after infection with the GFP virus (data not shown). (E) C57BL/6J mice were infected intranasally with WT virus, the EPI mutant, an independently derived EPI mutant (EPI-IND), or a revertant of the EPI virus (EPI-REV). Splenic infectious centres were then measured 13 and 17 d post-infection. The dashed line shows the lower limit of assay sensitivity.

### Antigen-Specific Immune Responses to the EPI Virus

The EPI virus was notably controlled without a need for the massive T cell activation that characterises MHV-68- or EBV-associated infectious mononucleosis (Figure 4D). We measured virus-specific immune responses (Figure 5) to gain some idea of what effector response might be responsible for the latency amplification deficit. At 13 d post-infection, ELISPOT assays (Figure 5A) showed a low CD4^+^ T cell response to the EPI virus compared to WT. A similar reduction in CD4^+^ T cell response is seen with MHV-68 specifically made to be latency deficient [42], presumably because lytic reactivation after latency amplification normally provides a large CD4^+^ T cell stimulus. Virus-specific serum antibody titres were marginally higher in the EPI-virus-infected mice (Figure 5B). CD8^+^ T cell responses to immunodominant MHV-68 lytic epitopes (p56 and p79) were comparable between WT and EPI viruses at day 13 post-infection (Figure 5A and 5C). Thus, the EPI virus was most likely being cleared by CTLs directed against an ORF73-associated epitope.

**Figure 5:**
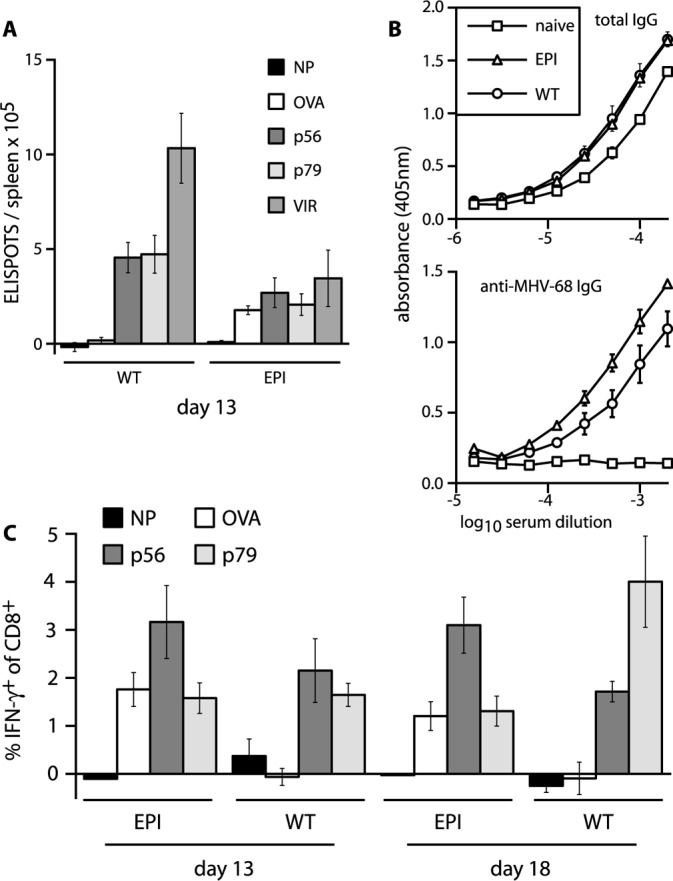
Antigen-Specific Immune Responses to the IRES-EPI Virus (A) CD8^+^ and CD4^+^ T cell responses were measured by interferon-gamma ELISPOT assay 13 d post-infection. The response to virus-exposed targets (VIR) is mediated by CD4^+^ T cells; the response to the p56, p79, and SIINFEKL (OVA) peptides is mediated by CD8^+^ T cells [62]. The mean number of spots with untreated targets was subtracted from the number of spots with each specific target. There was a response to the OVA peptide in the IRES-epitope construct, but not to the ASNENMETM peptide (NP). Mean ± SD values of five mice per group are shown. (B) Total and MHV-68 virion-specific serum IgG responses were measured by ELISA at 18 d post-infection. “Naive” indicates age-matched, uninfected controls. Mean ± SD absorbance values of four mouse sera per group are shown. (C) Spleen cells were stimulated for 5 h in the presence of Brefeldin A plus the peptide indicated and then stained for cell-surface CD8 and intracellular interferon-gamma. The percentage of interferon-gamma^+^ CD8^+^ cells without peptide was subtracted from the value with peptide to give the specific response. Mean ± SD values of five mice per group are shown.

There was no ASNENMETM-specific response to the EPI virus (Figure 5A and 5C). Also, there was no evidence of an enhanced response to the p79 epitope, which was present both in the IRES-epitope construct and in its native context in ORF61 [43]. These epitopes were probably not processed from the polytope construct, since transfecting it as an expression plasmid into H2-K^b^- or H2-D^b^-expressing L929 cells stimulated the SIINFEKL-specific hybridoma B3Z but not T cell hybridomas specific for ASNENMETM or p79 (data not shown). In contrast to the lack of response to ASNENMETM, there was a clear response to the SIINFEKL epitope co-expressed with ORF73 (Figure 5A and 5C), which was also presented in vitro (see Figure 3D). By 18 d post-infection (Figure 5C), the CD8^+^ T cell response to WT infection had made its characteristic shift in immunodominance from the p56 epitope associated with epithelial infection to the p79 epitope associated with B cell infection [43]. This did not occur with the EPI virus, presumably because the number of latently infected B cells remained low. Thus, it seemed likely that SIINFEKL-specific CTLs eliminated the EPI virus.

### Attenuation of the EPI Virus Is H2-Type-Restricted

As all of the CTL epitopes in the IRES-epitope construct were H2^b^-restricted, a major prediction was that the EPI virus would not be attenuated in H2^d^ mice. This was found to be the case (Figure 6). In H2^d^ BALB/c mice, the EPI virus attained infectious centre titres in the spleen equivalent to WT virus (Figure 6A). B cell activation (Figure 6B), splenomegaly (Figure 6C), and viral genome load (Figure 6D) were also normal. We further assayed latency by in situ hybridization for the expression of viral tRNA homologues in splenic germinal centres (Figure 6E). These are expressed at high levels in MHV-68-infected lymphoid tissue and provide an additional marker of latency establishment [44]. The EPI virus showed no viral tRNA^+^ cells in C57BL/6J mice and normal numbers in BALB/c mice. These data supported the idea that the attenuation of the EPI virus was due to the expression of a CTL target from the ORF73 mRNA.

**Figure 6:**
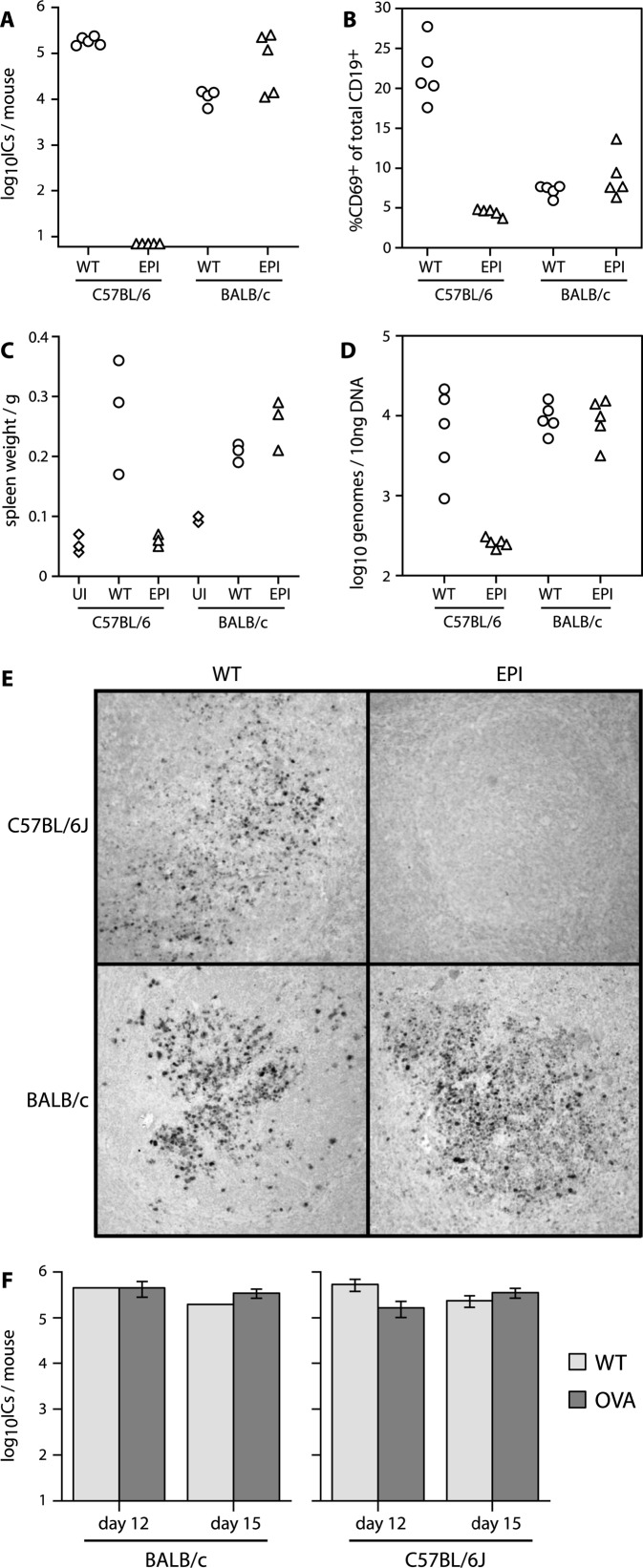
Normal EPI Virus Replication in Non-H2^b^ Mice (A) Infectious centre titres in individual spleens were determined 14 d after intranasal infection of C57BL/6J (H2^b^) or BALB/c (H2^d^) mice with WT or EPI virus. (B) CD69 expression on splenic B cells was measured by flow cytometry 14 d post-infection. B cells from uninfected mice were less than 5% CD69^+^. (C) The weights of individual spleens are shown 14 d post-infection, with spleens of age-matched, uninfected mice (UI) for comparison. (D) Viral genome loads in individual mice were determined by real-time PCR at 14 d post-infection. (E) Viral tRNA expression in infected germinal centres was visualised by in situ hybridization with a digoxigenin-labelled riboprobe specific for tRNAs 1–4. Representative follicles of at least five sections per mouse and three mice per group are shown. tRNA^+^ follicles were abundant with WT virus and with the EPI virus in BALB/c mice, but were not seen with the EPI virus in C57BL/6J mice. (F) BALB/c or C57BL/6J mice were infected intranasally with MHV-68 expressing OVA from an intergenic expression cassette (OVA) or with WT virus. The extent of lymphoid colonisation was determined by infectious centre assay of spleens 12 and 15 d post-infection. Mean ± SEM titres of five mice per group are shown. In contrast to the EPI virus, the OVA virus showed no defect in host colonisation.

In contrast to the EPI virus, MHV-OVA showed no significant attenuation in either BALB/c or C57BL/6J mice compared to WT virus (Figure 6F). MHV-OVA expresses the SIINFEKL epitope at high levels during lytic infection (see Figure 3D). Thus, SIINFEKL expression during episome maintenance, when epitopes are not normally presented, was catastrophic for the virus, whereas SIINFEKL expression outside of this context, when MHV-68 does not rely on limiting epitope presentation for its survival, had little effect.

### Attenuation of the EPI Virus Is CD8^+^ T Cell–Dependent

The MHC class I restriction of the EPI virus's latency deficit and its association with anti-SIINFEKL immunity implied that CD8^+^ T cells were eliminating latently infected cells. This was confirmed by rescuing the EPI virus with CD8^+^ T cell depletion (Figure 7). Thus, in C57BL/6J mice treated with an anti-CD8 monoclonal antibody, the EPI virus achieved WT levels of B cell activation (Figure 7A), viral genome load (Figure 7B), and infectious centres (Figure 7C). The ORF73 CTL evasion (see Figure 1) that was bypassed in the EPI virus was therefore essential for in vivo episome maintenance.

**Figure 7:**
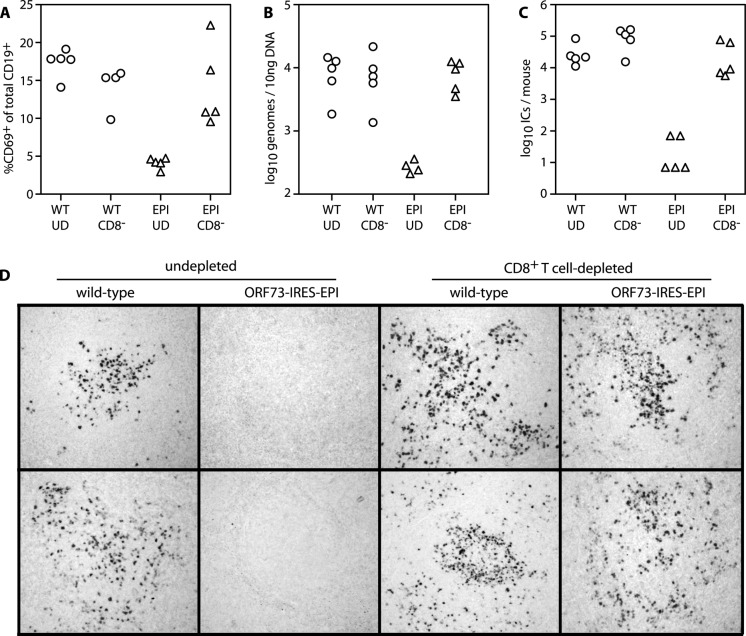
Rescue of the EPI Virus by CD8^+^ T Cell Depletion Mice were left undepleted (UD) or depleted of CD8^+^ T cells (CD8^−^) by an initial intravenous injection of mAb YTS169 2 d before infection, followed by intraperitoneal injections of the same antibody every 2–3 d up to the time of sampling. Infection was by intranasal inoculation of either WT or EPI viruses. (A) Depletion was 95%–99% complete as assessed by flow cytometry of spleen cells. CD69 expression on splenic B cells was measured 13 d post-infection. (B) Genome loads were measured 13 d post-infection by real-time PCR. Each point shows an individual mouse. (C) The infectious centre titres of individual mice at 13 d post-infection are shown for one of two equivalent experiments. The titres of pre-formed, infectious virus in freeze-thawed spleens were less than 5% of the infectious centre titres, so even after CD8^+^ T cell depletion, the infectious centre assay essentially measured latent virus. By 13 d post-infection, the lungs of both immunocompetent and CD8^+^ T cell–depleted mice were clear of infectious virus. (D) In situ hybridization for viral tRNA expression in splenic germinal centres is shown 13 d after infection of CD8^+^ T cell–depleted or undepleted mice, infected with either EPI or WT virus. Spleens of two representative mice are shown in each case.

## Discussion

The MHV-68 ORF73 lacks the glycine–alanine repeat of EBNA-1 but still conferred poor presentation on a linked CTL epitope. Thus, despite different means, MHV-68 and EBV have arrived at a similar end of inhibiting epitope presentation during episome maintenance. Neither molecular mechanism is fully understood, but both seem to rely primarily on limiting protein synthesis. A large proportion of CTL epitopes are derived from abortive translation events [45]. Understanding the mechanism of *cis-*acting CTL evasion therefore means understanding the major source of abortive translation events, whether damaged RNA, ribosomal errors, or protein misfolding. Since the key evasion regions of EBNA-1 and ORF73 are located centrally, they are translated only after potential N-terminal epitopes, and so may exert their inhibitory effects prior to translation, as RNA. Our aim here was to ask what *cis-*acting evasion contributes to the fitness of a gamma-herpesvirus. Inserting an IRES element downstream of ORF73 allowed us to bypass *cis-*acting immune evasion in MHV-68. CTLs then wiped out latency. We conclude that avoiding epitope presentation during episome maintenance is fundamental to gamma-herpesvirus survival.

As yet, no endogenous CTL epitopes to our knowledge have been described for the MHV-68 ORF73. This may reflect its *cis-*acting immune evasion in the same way that EBV infection was initially thought not to elicit EBNA-1-specific CTLs [46,47]. However, EBNA-1 epitopes can be presented by cross-priming [5]. It seems likely that MHV-68 will elicit ORF73-specific CTLs by a similar route. Certainly there is no lack of predicted cleavable, MHC class I–binding peptides in ORF73; for example, H2-D^b^, FSSTHPYTL; H2-K^b^, QCVTYYLL; H2-D^d^ and H2-K^d^, KYQGMRRHL; and H-L^d^, APPSPDVDV. Thus, evasion must occur at the level of endogenous ORF73 presentation.

The effectiveness of immune evasion is inevitably context-dependent. Defining its impact on host colonisation therefore requires natural thresholds of in vivo antigen presentation. The results are not always predictable. For example, MHV-68 transcribes its K3 gene in the lytic cycle as well as in latency, but a lack of K3 has no discernable impact on primary lytic infection, only on latency amplification [31]. The recognition of EBNA-1 during latency III [7,8,9] does not necessarily imply EBNA-1 recognition during latency I, when autoregulation [48] and a cell cycle dependence [49] of the Qp promoter reduce EBNA-1 transcription. Our results with MHV-68 suggest that the EBNA-1 glycine–alanine repeat is a key component of in vivo EBV persistence. Of course MHV-68 is not EBV, and it is possible that the expression of a strong MHC class I–binding peptide exaggerated somewhat the potential of ORF73-specific CTLs to control infection. However, a clear message is that *cis-*acting CTL evasion is an important feature of the gamma-herpesvirus lifecycle.

Latency-associated *trans*-acting CTL evasion comes into play during the MHV-68 growth program, when rapid cell division probably raises ORF73 production above a level that can be disguised by *cis-*acting evasion, and additional viral gene products are expressed. This *trans-*acting evasion allows latency amplification to progress despite evidence of a CTL response to at least one viral growth program antigen [32]. However, *trans*-acting evasion alone was insufficient for even an initial amplification of MHV-68 latency. SIINFEKL production from the EPI virus did not simply compromise K3 function, since a complete loss of K3 typically gives a 1-log reduction in infectious centres with relatively little effect on the viral genome load [31], whereas the EPI virus showed a 3- to 4-log infectious centre deficit and a severely reduced genome load. *cis*-acting immune evasion therefore operated in a distinct setting relatively early in latency establishment. Multiple patterns of both EBV [50,51] and MHV-68 [14] latent gene expression occur in acutely infected lymphoid tissue. Notably, EBV implements EBNA-1-only latency even during acute infectious mononucleosis [35]. Thus, the extreme dependence of MHV-68 on *cis-*acting evasion probably reflects early B cell entry into “ORF73-only” latency.

The rather modest SIINFEKL-specific response to the EPI virus contrasted with the massive CTL activation stimulated by WT virus. For example, in one experiment WT virus progressed from 4.7 × 10^3^ infectious centres per spleen at day 10 of infection to 6.4 × 10^4^ at day 14 (means of five mice), while the percentage of CD8^+^ T cells expressing CD69 increased from 10.7% to 20.8%. Over the same time, the EPI virus infectious centres fell from 7.3 × 10^2^ to 4.6 × 10^1^, with 6.9% and 5.9%, respectively, of CD8^+^ T cells expressing CD69. Indeed the numerous lytic antigen-specific CTLs stimulated by EBV [52] and MHV-68 [43] infections imply an immune response failure, since latently infected B cells proliferate and progress to lytic gene expression without hindrance by latent antigen-specific CTLs. It is crucial in persistent viral infections for the immune system to attack appropriate targets. End-stage cells may stimulate large T cell responses, but the control of infection depends more on overcoming immune evasion. A major challenge in vaccinating against complex pathogens is to direct the immune system against the key, self-renewing population that maintains the parasite load.

## Materials and Methods

### 

#### Mice

C57BL/6J and BALB/c mice (Harlan Olac, Bicester, United Kingdom) were kept in Cambridge University animal facilities in accordance with United Kingdom Home Office guidelines (project licence 80/1579). Mice were infected intranasally with 2 × 10^4^ plaque-forming units (PFU) of MHV-68 under brief halothane anaesthesia. T cell subset depletion was by intravenous and then intraperitoneal injection of purified mAb YTS169 [53].

#### Cell lines

BHK-21 cells, MEF-1 cells, NIH-3T3-CRE cells [31], A20 cells, L929 cells transfected with H2-K^b^ [54], and the B3Z T cell hybridoma [55 were all grown in DMEM, supplemented with 2 mM glutamine, 100 U/ml penicillin, 100 μg/ml streptomycin, and 10% fetal calf serum (complete DMEM). A20-syndecan-1 cells were made by retroviral transduction of A20 cells with a vector expressing the extracellular domain of murine syndecan-1 linked to the transmembrane and cytoplasmic domains of H2-D^b^, and will be described in detail elsewhere. MEFs were harvested at 13 d of gestation and were grown in complete DMEM with 50 μM 2-mercaptoethanol.

#### Plasmids

We amplified ORF73 by PCR (Hi-Fidelity PCR kit, Roche Diagnostics, Lewes, United Kingdom), including EcoRI and SalI restriction sites at its respective 5′ and 3′ ends, and cloned the product into the EcoRI and SalI sites of pSP73 (Promega, Chilworth, United Kingdom) to make pSP73-ORF73. To introduce the SIINFEKL epitope of OVA [56] near the 3′ end of ORF73, we digested pSP73-ORF73 with HinDIII and dephosphorylated it (P. borealis alkaline phosphatase, Roche Diagnostics). Two complementary oligonucleotides (5′- AGCTAGTATAATCAACTTTGAAAAACTGCT and 5′- AGCTAGCAGTTTTTCAAAGTTGATTATACT) (Sigma-Genosys, Cambridge, United Kingdom) were then heated, annealed, phosphorylated, and ligated into the HinDIII site (T4 DNA ligase, New England Biolabs, Hitchin, United Kingdom). Thus, amino acid residues 277–283 of ORF73 (QASGTQH) were changed to QASIINFEKLLASGTQH (ORF73-SC). Oligonucleotide insertion was confirmed by DNA sequencing. To insert the SIINFEKL coding sequence in the 5′ end of ORF73, we amplified ORF73 by PCR, pairing the 5′ primer 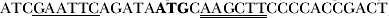, containing a 5′ EcoRI restriction site (underlined) upstream and a HinDIII site (double-underlined) downstream of the ORF73 start codon (bold), with a 3′ primer downstream of the ORF73 BstEII site, and containing an XhoI site. This PCR product was cloned into the EcoRI and XhoI sites of pSP73. The complementary oligonucleotides 5′- AGCTAGTATAATCAACTTTGAAAAACTGAC and 5′- AGCTGTCAGTTTTTCAAAGTTGATTATACT were then inserted into the HinDIII site, changing amino acid residues 1–6 of ORF73 from MPTSPP to MQASIINFEKLTASPP (ORF73-SN). The modified 5′ end of ORF73 was then subcloned as an EcoRI/BstEII fragment into pSP73-ORF73, thereby reconstituting the gene with its 3′ HinDIII site intact. Each form of ORF73 was then cloned into the pcDNA3 mammalian expression vector (Invitrogen, Carlsbad, California, United States). We made 3′ deletions of pcDNA3-ORF73-SN by digesting it with HinDIII or KpnI, each of which cuts within ORF73 and within the pcDNA3 polylinker 5′ of its EcoRI site. The N-terminal ORF73 fragment was then gel-purified and ligated into a new pcDNA3 vector. We generated a 3′ PstI deletion by digestion with PstI, gel purification, and ligation of the vector back to itself, since PstI cuts downstream of the pcDNA3 XhoI site.

We subcloned the N-terminal 325 amino acid residues of OVA as an EcoRI/XhoI fragment from pMSCV-OVA-IRES-GFP [57] into pSP73. Hybrids of 5′ ORF73 and 3′ OVA (which contains the SIINFEKL epitope), or 5′ OVA and 3′ ORF73 (with its SIINFEKL insert), were made by cutting each at a unique internal PstI site and swapping an in-frame 3′ PstI/XhoI fragment between them. Each form of ORF73/OVA was then subcloned as an EcoRI/XhoI fragment into the EcoRI and XhoI sites of pcDNA3. To fuse the ORF73 coding sequence to the C-terminus of OVA, we PCR-cloned the N-terminal 325 amino acid residues of OVA without a stop codon into the EcoRI and XhoI sites of pcDNA3 and ligated in PCR-cloned ORF73 as an XhoI/ApaI fragment, downstream of and in frame with the OVA coding sequence. 3′ HinDIII and KpnI truncations of this construct were generated as above. We also generated 5′ ORF73 truncations as XhoI/ApaI-digested PCR products, starting at amino acid residue 126 (OVA-73E), 146 (OVA-73D), 166 (OVA-73C), 186 (OVA-73B), or 206 (OVA-73A), and fused these to OVA 1–325 in pcDNA3. We also cloned OVA lacking its signal sequence, with translation starting at its methionine residue 41 (SOVA), and made the same fusions with N-terminal ORF73 truncations A–E.

#### Recombinant viruses

The MHV-68 M11 and ORF73 coding sequences (genomic co-ordinates 103418–103933 and 104868–103924, respectively) overlap by 10 bp at their 3′ ends [11]. We therefore duplicated this overlap to generate an insertion site between them. Thus, we PCR-cloned genomic co-ordinates 131171–103933, including XhoI and SalI restriction sites at the respective 5′ and 3′ ends, and cloned the fragment into the XhoI and SalI sites of pSP73-ORF73 (see Plasmids, above) to make pSP73-ORF73-M11, with the M11 and ORF73 coding sequences each complete and separated by a SalI site. The M11/ORF73 genomic overlap 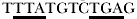 (the stop codons of ORF73 on the noncoding strand and M11 on the coding strand are underlined) was thus changed to TTTATGTC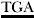 GTCGACTTATGTCTGAG. We then generated a poly-epitope construct downstream of an encephalomyocarditis IRES for insertion into the SalI site. We first inserted the adenovirus E19K leader sequence as two complementary oligonucleotides (5′- AATTGACCACCATGAGGTACATGATTTTAGGCTTGCTCGCCCTTGCGGAGTCTGCAGCGCGAATTCAGATCTCTCGAGTGAT and 5′- TCGAATCACTCGAGAGATCTGAATTCGCGCTGCAGACTGCCGCAAGGGCGAGCAAGCCTAAAATCATGTACCTCATGGTGGTC) into the EcoRI and XhoI sites of pMSCV-IRES-NEO [57]. Two complementary oligonucleotides encoding the peptide sequence MTSINFVKIASNENMETMSIINFEKL (5′- AATTCCTACCACCATGACCAGTATCAACTTTGTGAAGATAGCTTCCAATGAAAACATGGAGACTATGAGTATAATCAACTTTGAAAAACTGTGAC and 5′- TCGAGTCACAGTTTTTCAAAGTTGATTATACTCATAGTCTCCATGTTTTCATTGGAAGCTATCTTCACAAAGTTGATACTGGTCATGGTGGTAG) were then inserted into the EcoRI and XhoI sites of the pMSCV-NEO-leader construct. TSINFVKI is an H2-K^b^-restricted epitope from the MHV-68 ORF61 [43]; ASNENMETM is an H2-D^b^-restricted epitope from the influenza A/PR/8/34 nucleoprotein [58]. We amplified the leader-epitope construct by PCR, using the primers 5′- CCCCCATGGCCAGGTACATGATTTTAGGCTTGCTC and 5′- CCCGTCGACTCACAGTTTTTCAAAGTTGATTATACT, thereby adding a 5′ NcoI site and replacing the 3′ XhoI site with a 3′ SalI site, and cloned it into the NcoI and SalI sites of pMSCV-IRES-GFP [59]. Thus, the GFP coding sequence downstream of the IRES was replaced by the leader-epitope construct. We used the 3′ SalI site and an XhoI site just 5′ of the IRES to excise an IRES-leader-epitope XhoI/SalI fragment and cloned it into the SalI site of pSP73-ORF73-M11. We also subcloned an XhoI/SalI IRES-GFP fragment from pMSCV-IRES-GFP into the SalI site of pSP73-ORF73-M11 to make a control virus. Each ORF73-IRES construct was then subcloned into a larger genomic fragment for recombination into the MHV-68 genome. To do this, we used a BamHI-G genomic fragment [10] (genomic co-ordinates 101653–106902), cloned into pACYC184 (New England Biolabs) lacking a BspHI site [60]. The ORF73-IRES constructs and pACYC184-BamHI-G were digested with BstEII (genomic co-ordinate 104379) and BspHI (genomic co-ordinate 103750). Because BspHI is blocked by methylation, we used plasmids derived from Dam^−^ *E. coli.* Finally, the mutant BamHI-G fragments were subcloned into the BAC mutagenesis shuttle vector pST76K-SR. Rec A–mediated recombination into the MHV-68 BAC was then carried out as previously described [41]. Sequence analysis revealed that the E19K leader sequence had been mutated during cloning, destroying the NcoI site and changing the start of the coding sequence downstream of the IRES from MARYMILG to MILG. Since this change was unlikely to prevent epitope presentation, no attempt was made to correct it. The ORF73-IRES-epitope BAC was subsequently reverted using an unmutated BamHI-G clone in pST76K-SR. MHV-OVA was generated by cloning OVA cDNA into EcoRI and XhoI sites of an ORF57/ORF58 intergenic expression cassette, driven by an ecotopic MHV-68 M3 promoter [61]. This virus will be described in more detail elsewhere. All BACs were reconstituted into infectious virus by transfecting 5 μg of BAC DNA into BHK-21 cells with Fugene-6 (Roche Diagnostics). The BAC cassette was removed by serial viral passage through NIH-3T3-CRE cells. Virus stocks were grown and titred on BHK-21 cells.

#### Virus assays

Infectious virus in freeze-thawed lung and spleen homogenates was plaque assayed on MEFs. Latent plus pre-formed virus in spleens was assayed on MEFs by explant culture of single-cell suspensions [39]. Cells expressing viral tRNAs 1–4 were detected by in situ hybridization of formalin-fixed, paraffin-embedded spleen cell sections, using a digoxigenin-labelled riboprobe transcribed from pEH1.4 [44]. Hybridized probe was detected with alkaline phosphatase-conjugated anti-digoxigenin Fab fragments (Boehringer Ingelheim, Bracknell, United Kingdom) according to the manufacturer's instructions. The viral genome load in individual spleens was measured by real-time PCR. DNA was extracted (Wizard genomic DNA purification kit, Promega) and a portion of the MK3 ORF (genomic co-ordinates 24832–25071) amplified by PCR from 10 ng of each sample (Rotor Gene 3000, Corbett Research, Cambridge, United Kingdom). PCR products were quantitated with Sybr green (Invitrogen) and compared with a standard curve of cloned MK3 template, serially diluted in uninfected cellular DNA and amplified in parallel. The MK3 copy number was calculated from the cycle number at which the Sybr green signal crossed a set threshold on the standard curve. Amplified products were distinguished from paired primers by melting curve analysis, and the correct size of the amplified products was confirmed by electrophoresis and staining with ethidium bromide.

#### Southern blotting

Viral DNA was isolated from infected BHK-21 cells by alkaline lysis [39], digested with NcoI, electrophoresed on a 0.8% agarose gel, and transferred to positively charged nylon membranes (Roche Diagnostics). A ^32^P-dCTP-labelled probe (APBiotech, Amersham, United Kingdom) was generated from the BamHI-G genomic fragment by random primer extension (Nonaprimer kit, Qbiogene, Bingham, United Kingdom) according to the manufacturer's instructions. Membranes were hybridized with probe (65 °C, 18 h), washed to a stringency of 0.2× SSC with 0.1% SDS, and exposed to X-ray film.

#### Metabolic labelling and immunoprecipitation

Cells were metabolically pulse-labelled with ^35^S-cysteine/methionine (APBiotech) and chased with 1 mM unlabelled cysteine and methionine [29]. Labelled cells were lysed on ice for 30 min in 50 mM Tris-Cl (pH 7.4), 150 mM NaCl, 5 mM EDTA, 1% Triton X-100, 1 mM PMSF, plus Complete protease inhibitors (Roche Diagnostics). Cell debris and nuclei were removed by centrifugation (13,000 × *g,* 15 min). Lysates were precleared with rabbit anti-actin whole serum and formalin-fixed S. aureus (Sigma Chemical, Poole, United Kingdom), and then again with protein A-sepharose. OVA was precipitated with rabbit anti-OVA serum (Abcam, Cambridge, United Kingdom) followed by protein A-sepharose. Beads were washed five times in 1% Triton X-100 buffer. Samples were dissociated (95 °C, 2 min) in Laemmli's buffer prior to SDS-PAGE. Gels were fixed, dried, and exposed to X-ray film.

#### Immunoblotting

Cells were lysed as for immune precipitations (above). Post-nuclear lysates were denatured (95 °C, 2 min) in Laemmli's buffer, separated by SDS-PAGE, and transferred to polyvinylidene difluoride membranes. Membranes were blocked in PBS/0.1% Tween-20/10% non-milk fat and probed with rabbit anti-OVA serum or rabbit anti-neomycin phosphotransferase II serum (Upstate, Milton Keynes, United Kingdom), followed by horseradish-peroxidase-coupled donkey anti-rabbit IgG pAb (APBiotech) and ECL substrate development.

#### Antigen presentation assays

L929-K^b^ cells (1–2 × 10^5^/well in 24-well plates) were transfected with 1 μg of plasmid using Fugene-6. Forty-eight hours later, B3Z cells (5 × 10^5^) were added to each well. B3Z is an H2-K^b^-restricted, SIINFEKL-specific T cell hybridoma that produces beta-galactosidase in response to T cell receptor ligation [55]. For virus infections, L929-K^b^ cells or MEF-1 cells (5 × 10^5^/well) were infected for 2 h and washed once before adding B3Z T cell hybridoma cells (5 × 10^5^/well). After a further 18 h, the cells were washed once in PBS and lysed in PBS/5 mM MgCl_2_/1% NP-40/0.15 μM chlorophenol-red-beta-D-galactoside (Merck Biosciences, Nottingham, United Kingdom) to assay beta-galactosidase activity. After 2–4 h at 37 °C, the absorbance at 595 nm was read on a Bio-Rad (Hercules, California, United States) Benchmark microplate reader.

#### ELISA and ELISPOT assays

For IFN-γ ELISPOT assays [62], duplicate dilutions of effector cells were incubated with 3 × 10^5^ naive irradiated syngeneic spleen cells in nitrocellulose-bottomed 96-well plates (Millipore Corporation, Bedford, Massachusetts, United States) coated with rat anti-mouse IFN-γ mAb (BD-Pharmingen, San Diego, California, United States). The naive spleen cells were either (1) untreated, (2) pulsed with 1 μM AGPHNDMEI (p56), 1 μM TSINFVKI (p79), 1 μM ASNENMETM, or 1 μM SIINFEKL peptide, or (3) infected with WT MHV-68 (2 PFU/cell). After 48 h culture at 37 °C in complete RPMI/50 μM 2-mercaptoethanol/10 U/ml human recombinant IL-2, captured IFN-γ was detected with a further, biotinylated rat anti-mouse IFN-γ mAb (BD-Pharmingen), followed by streptavidin-alkaline phosphatase (Dako Cytomation, Ely, United Kingdom) and 5-bromo-4-chloro-3-indolyl phosphate/nitro blue tetrazolium substrate.

Virus-specific and total serum IgG levels were measured by ELISA. Maxisorp ELISA plates (Nalge Nunc, Rochester, New York, United States) were coated overnight with either affinity-purified goat anti-mouse IgG sera (Sigma Chemical) or 0.05% Triton X-100–disrupted MHV-68 [63]. After incubation with 2-fold serum dilutions, bound murine IgG was detected with alkaline phosphatase-conjugated goat anti-mouse IgG–Fcγ serum and nitrophenylphosphate substrate (Sigma Chemical). Absorbance was read at 405 nm.

#### Flow cytometry

A20-syndecan-1 cells infected with GFP^+^ viruses were trysinized, washed in PBS, and analysed directly for green channel fluorescence. Spleens were disrupted into single-cell suspensions, washed in PBS/0.1% BSA/0.01% azide, and incubated for 15 min on ice with 5% mouse serum/5% rat serum and anti-CD16/32 mAb. Specific staining (1 h, 4 °C) was with fluorescein-isothiocyanate-coupled anti-CD69 and phycoerythrin-coupled anti-CD19 (BD-Pharmingen), or tricolour-coupled anti-CD8 (Caltag Laboratories, Burlingame, California, United States), Fluorescein-isothiocyanate-coupled anti–T cell receptor Vbeta4, and phycoerythrin-coupled anti-CD62L (BD-Pharmingen). For intracellular cytokine staining, spleen cells (5 × 10^5^–1 × 10^6^ in 200 μl of complete RPMI/50 μM 2-mercaptoethanol/10 U/ml human recombinant IL-2/10 μg/ml Brefeldin A) were stimulated (5 h, 37 °C) with 1 μM ASNENMETM, 1 μM AGPHNDMEI, 1 μM TSINFVKI, or 1 μM SIINFEKL peptides, or left without peptide. All cells were then washed in PBS/10 μg/ml Brefeldin A, blocked with anti-CD16/32, stained with tricolour-conjugated anti-CD8 plus fluorescein-isothiocyanate-conjugated anti-I-A^b^ (1 h, 4 °C), washed twice, fixed in 2% paraformaldehyde (30 min, 4 °C), washed once, permeabilized with 0.5% saponin, washed once, stained with phycoerythrin-coupled anti-interferon-gamma (BD-Pharmingen), and washed twice. All cells were analysed on a FACS Calibur using Cellquest software (Becton-Dickinson, Oxford, United Kingdom). I-A^b^ staining was used to exclude B cells and myeloid cells. Data were graphed with FCSPress v1.3 (www.fcspress.com).

## Abbreviations

CTL: CD8^+^ T lymphocyte
EBV: Epstein-Barr virus
EPI: ORF73-IRES-epitope
GFP: green fluorescent protein
IRES: internal ribosome entry site
MEF: murine embryonic fibroblast
MHC: major histocompatibility complex
MHV-68: murine gamma-herpesvirus-68
OVA: ovalbumin
PFU: plaque-forming units
SD: standard deviation
WT: wild-type
